# Endoscopic closure of a refractory urethroanal fistula using an innovative wound closure device

**DOI:** 10.1055/a-2194-0305

**Published:** 2023-12-21

**Authors:** Pierre Lafeuille, Jérôme Rivory, Alexandru Lupu, Elena de Cristofaro, Jean-Christophe Saurin, Florian Rostain, Mathieu Pioche

**Affiliations:** 1 Department of Endoscopy and Hepatogastroenterology, Pavillon L, Edouard Herriot Hospital, Lyon, France; 2 Department of Systems Medicine, Gastroenterology and Endoscopy Unit, Tor Vergata University of Rome, Rome, Italy

Gastrointestinal (GI) fistula is a rare chronic disease that affects the quality of life of patients and represents a real therapeutic challenge, with frequent recurrences 1 . Endoscopic management of GI fistulas combines both endoscopic submucosal dissection (ESD) and mechanical closure of the orifice 2 . We recently reported that the strategy of fistula endoscopic submucosal dissection with clip closure (FESDC) is effective and safe for permanent closure of GI fistulas 3 , including in the exceptional cases where an aortoesophageal fistula has occurred 4 . In the case of anal fistulas, mechanical closure remains the greatest challenge because of the proximity of the anal sphincter, which does not allow effective closure using standard or over-the-scope clips.

We herein report the case of a 29-year-old man referred for a refractory urethroanal fistula with an existing intermediate imperforate anus requiring multiple surgical and endoscopic procedures.

We first performed the endoscopic examination without anesthetic. The fistula was visualized on the posterior wall of the anal verge during micturition by the patient, the bladder having first been filled with blue dye using a urinary catheter ( Fig. 1 ). After ESD of the internal orifice of the fistula, we decided to use the new Sutuart flexible needle holder (Olympus, Tokyo, Japan) 5 with a barbed suture (Medtronic, USA) to suture together the edges of the dissected fistula tract ( Fig. 2 , Video 1 ). This novel device allowed us to suture under endoscopic control in a tight area using barbed suture ( Fig. 3 ). Technical success was achieved, defined by tight sealing of the orifice confirmed by opacification at the end of the procedure without any urine leakage ( Fig. 4 ). No adverse event was reported. Suturing techniques with this new needle holder could be added to the range of existing closure methods after ESD of gastrointestinal fistulas.

**Fig. 1:**
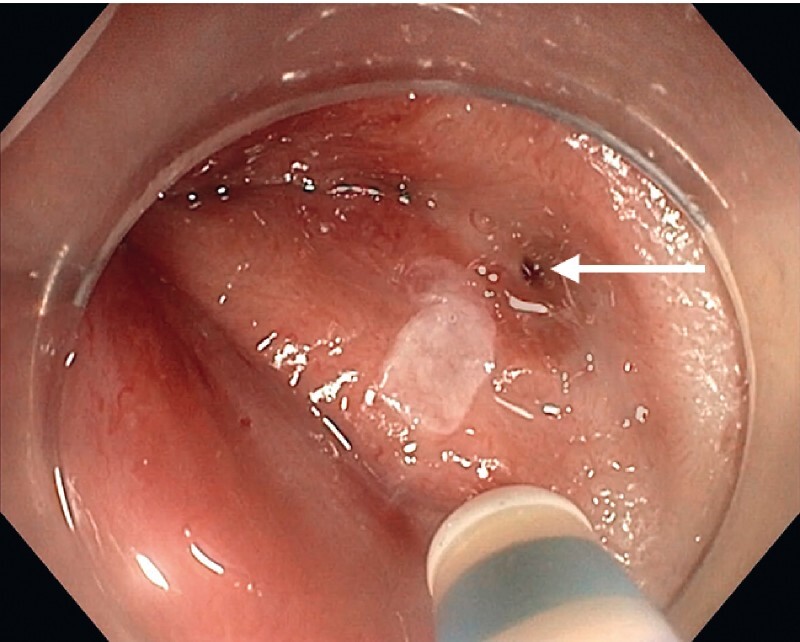
Endoscopic view of a urethroanal fistula (arrow).

**Fig. 2:**
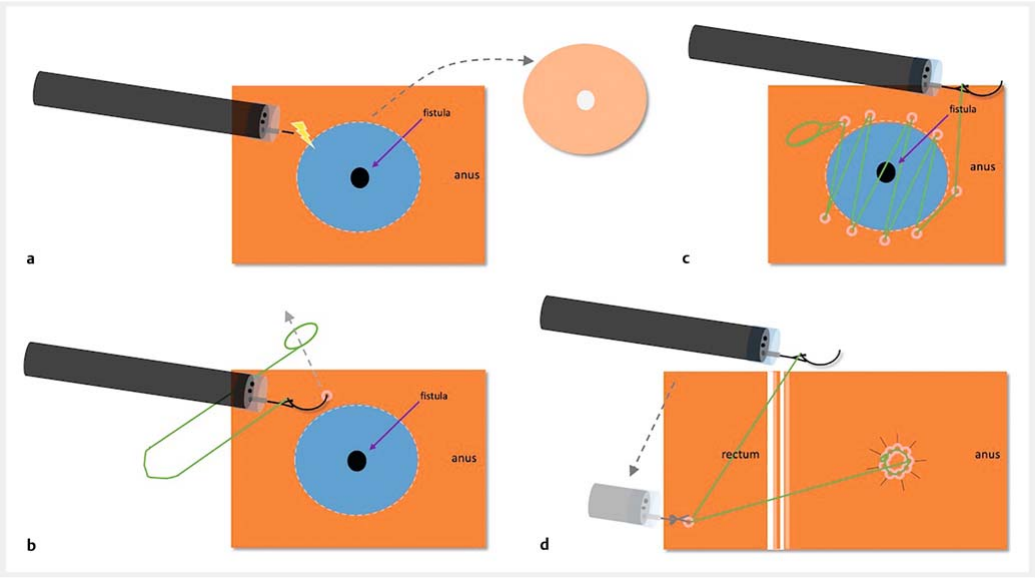
Schematic of endoscopic closure of the refractory urethroanal fistula using an innovative wound closure device (view from above). **a** The mucosal flap is completely dissected. **b** The needle is passed through the preformed anchored loop to begin apposition of the edges of the fistula orifice. **c** The edges of the fistula orifice are further approximated using a continuous suture path. **d** The device is anchored in the rectum and the suture finally cut.

**Video 1** Endoscopic closure of a refractory urethroanal fistula using an innovative wound closure device.


**Fig. 3 a:**
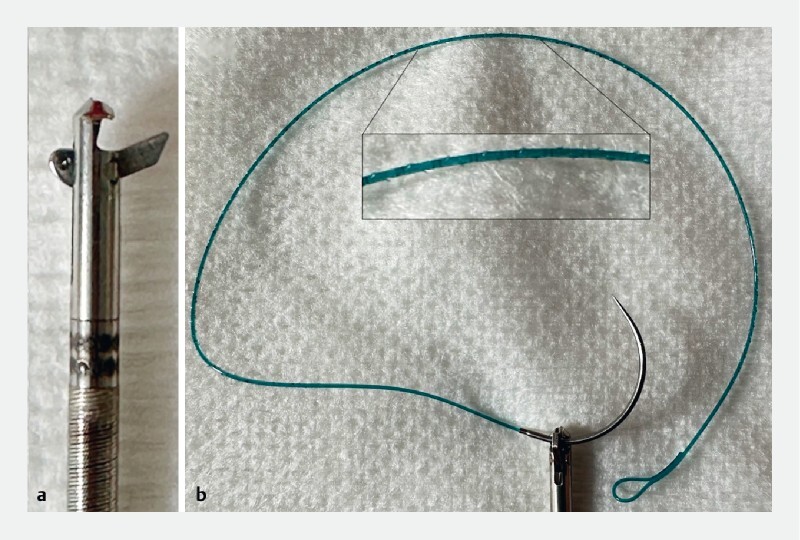
Sutuart needle holder in the open position. **b** V-Loc wound closure device with dual-angle cut and barbing pattern, in position in the needle holder.

**Fig. 4:**
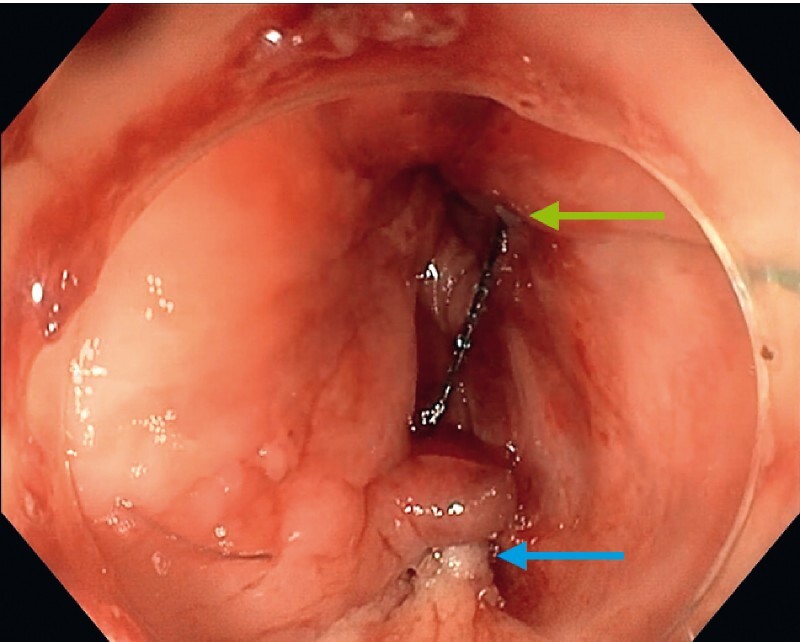
Endoscopic view of the closed fistula after the procedure: the edges of the dissected fistula orifice have been apposed (blue arrow) and anchored in the rectum (green arrow) – endoscopic view.

Endoscopy_UCTN_Code_TTT_1AQ_2AG

Citation Format Endoscopy 2023; 55: E1105–E1107. doi: 10.1055/a-2177-3695 .

## Notes

Endoscopy E-Videos

https://eref.thieme.de/e-videos
*E-Videos* is an open access online section of the journal *Endoscopy* , reporting on interesting cases and new techniques in gastroenterological endoscopy. All papers include a high-quality video and are published with a Creative Commons CC-BY license. Endoscopy E-Videos qualify for HINARI discounts and waivers and eligibility is automatically checked during the submission process. We grant 100% waivers to articles whose corresponding authors are based in Group A countries and 50% waivers to those who are based in Group B countries as classified by Research4Life (see: https://www.research4life.org/access/eligibility/ ). This section has its own submission website at https://mc.manuscriptcentral.com/e-videos
